# Evaluation of ^18^F-nifene binding to α4β2 nicotinic receptors in the rat brain using microPET imaging

**DOI:** 10.1186/2191-219X-1-6

**Published:** 2011-06-20

**Authors:** Ritu Kant, Cristian C Constantinescu, Puja Parekh, Suresh K Pandey, Min-Liang Pan, Balu Easwaramoorthy, Jogeshwar Mukherjee

**Affiliations:** 1 Preclinical Imaging Center, Department of Psychiatry and Human Behavior, University of California-Irvine, Irvine, CA 92697, USA

## Abstract

MicroPET imaging studies using ^18^F-nifene, a new positron emission tomography (PET) radiotracer for nicotinic acetylcholinergic receptors (nAChR) α4β2 receptors in rats, have been carried out. Rats were imaged for 90 min after intravenous injection of ^18^F-nifene (0.8 to 1 mCi), and binding potential (BP_ND_) was measured. ^18^F-Nifene binding to thalamic and extrathalamic brain regions was consistent with the α4β2 nAChR distribution in the rat brain. Using the cerebellum as a reference, the values for the thalamus varied less than 5% (BP_ND _= 1.30, *n *= 3), confirming reproducibility of ^18^F-nifene binding. ^18^F-Nifene microPET imaging was also used to evaluate effects of nicotine in a group of Sprague-Dawley rats under isoflurane anesthesia. Nicotine challenge postadministration of ^18^F-nifene demonstrated reversibility of ^18^F-nifene binding *in vivo*. For α4β2 nAChR receptor occupancy (nAChR_OCC_), various doses of nicotine (0, 0.02, 0.1, 0.25, and 0.50 mg/kg nicotine free base) 15 min prior to ^18^F-nifene were administered. Low-dose nicotine (0.02 mg) reached > 80% nAChR_OCC _while at higher doses (0.25 mg) > 90% nAChR_OCC _was measured. The small amount of ^18^F-nifene binding with reference to the cerebellum affects an accurate evaluation of nAChR_OCC_. Efforts are underway to identify alternate reference regions for ^18^F-nifene microPET studies in rodents.

## Background

**Figure 1:**
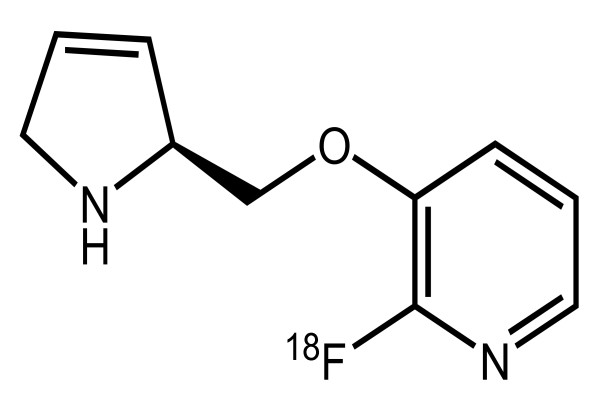
**Chemical structure of ^18^F-nifene**.

Nicotinic α4β2 receptors play an important role in many CNS disorders such as Alzheimer's disease, Parkinson's disease, Schizophrenia, mood disorders, and nicotine dependence. Much work is being done on radiotracer compounds with high binding affinity as well as faster kinetics which can be used as an aid to visualize the nicotinic receptors and their involvement in neurological disorders [1]. Both 5-^123^I-iodo-A-85380 and 2-^18^F-fluoro-A-85380 have a high affinity for the α4β2 receptors with scan times exceeding several hours. In order to reduce the scan time, emphasis was placed on developing a tracer with faster kinetics. We have developed ^18^F-nifene (2-^18^F-fluoro-3-[2-((S)-3-pyrrolinyl)methoxy]pyridine; Figure 1), a nicotinic α4β2 receptor agonist which is suitable for positron emission tomography (PET) imaging (*K*_i _= 0.50 nM; [2,3]). Imaging times in nonhuman primates with ^18^F-nifene [2] were reduced significantly compared to ^18^F-flouroA-85380 [4].

Nicotine has a high affinity for α4β2 nicotinic acetylcholinergic receptors (nAChR) receptors (*K*_i _= 1.68 nM, [3]). Cigarette smoking and nicotine (a major component of tobacco) have been shown to have a direct and significant occupancy of α4β2 nAChR receptors [5-7]. Studies have also shown an increase in α4β2 receptor density binding sites in rat and mice brains upon exposure to nicotine [8-10]. Chronic tobacco smoking increases the number of high affinity nAChRs in various brain areas [11]. Human postmortem data have shown the presence of α4β2 nAChR receptors in the subiculum, which are upregulated in smokers [10]. Human imaging studies, using SPECT imaging agent 5-^123^I-iodo-A-85380 and PET imaging agent 2-^18^F-fluoro-A-85380, have also identified an increase in receptor density among smokers versus nonsmokers, suggesting 2-^18^F-fluoro-A-85380 to be a reliable PET method for further tobacco studies [12,13]. As reported recently, nicotine from typical cigarette smoking by daily smokers is likely to occupy a majority of α4β2 receptors and lend them to a desensitized state [5]. Thus, noninvasive imaging is playing a major role in understanding nicotine dependency [14,15].

The focus in this work is on *in vivo *evaluation of ^18^F-nifene binding to α4β2 nicotinic receptors in rodent brain regions using microPET. In an effort to establish ^18^F-nifene microPET studies in the rat model, our objectives were the following: (1) evaluate *in vivo *^18^F-nifene in the normal rat model using microPET and confirm by *ex vivo *microPET and autoradiography, (2) carry out test-retest microPET studies in the rat model in order to evaluate reproducibility of ^18^F-nifene microPET binding, and (3) measure changes in ^18^F-nifene binding in the rat model using microPET at different doses of nicotine. These findings will assist in our eventual goal to evaluate the role of α4β2 nAChR in nicotine dependency using the rodent model.

## Methods

### General methods

All chemicals and solvents were purchased from Aldrich Chemical (Aldrich Chemical Company, Wilwaukee, WI, USA) and Fisher Scientific (Fisher Scientific UK Ltd., Leicestershire, UK). Deionized water was acquired from Millipore Milli-Q Water Purification System (Millipore, Billerica, MA, USA). Gilson high-performance liquid chromatography (HPLC) was used for the semipreparative reverse phase column chromatography. Fluorine-18 fluoride was produced via MC-17 cyclotron using oxygen-18-enriched water. Radioactivity was counted using a Capintec dose calibrator while low level counting was done using a well counter. Inveon preclinical Dedicated PET (Siemen's Inc., Munich, Germany) was used for the microPET studies which has a resolution of 1.45 mm [16]. Both *in vivo *and *ex vivo *images of the rat brains were obtained using the Inveon microPET scanner and were analyzed using the Acquisition Sinogram Image Processing (ASIPRO, Siemens Medical Solutions USA, Inc., Knoxville, TN, USA) and Pixelwise Modeling Software (PMOD Technologies, Zurich, Switzerland). Slices of the rat brain were prepared at 10 to 40-μm thick using the Leica 1850 cryotome (Leica Instruments, Nussloch, Germany). *In vitro*- or *ex vivo*-labeled brain sections were exposed to phosphor films (Perkin Elmer Multisensitive, Medium MS) and were read using the Cyclone Phosphor Imaging System (Packard Instruments, Meriden, CT, USA). An analysis of *in vitro *or *ex vivo *autoradiographs was done using the Optiquant Acquisition and Analysis software (Packard Instruments, Meriden, CT, USA). All animal studies have been approved by the Institutional Animal Health Care and Use Committee of the University of California, Irvine.

### Radiolabeling

A synthesis of ^18^F-nifene was carried out following reported procedures (Pichika et al. 2006). The automated radiosynthesis of ^18^F-nifene was carried out in the chemistry processing control unit box. An Alltech C_18 _column (10 μm, 250 × 10 mm^2^) was used for reverse phase HPLC purification and specific activity of ^18^F-nifene was approximately 2,000 Ci/mmol.

### MicroPET ^18^F-nifene studies

Male Sprague-Dawley rats were fasted 24 h prior to the time of scan. On the day of the study, rats were anesthetized using 4.0% isoflurane. The rat was then positioned on the scanner bed by placing it on a warm water circulating heating pad, and anesthesia was applied using a nose cone. A transmission scan was subsequently acquired. The preparation of the dose injection was as follows: 0.7-1.0 mCi of ^18^F-nifene was drawn into a 1-mL syringe with a 25-gauge needle and was diluted with sterile saline to a final volume of 0.3 mL. The dose was injected intravenously into the tail vein of the rat. Isoflurane was reduced and maintained at 2.5% following the injection. The scans were carried out for 90 min and were acquired by the Inveon microPET in full list mode. The list mode data were collected dynamically which were rebinned using a Fourier rebinning algorithm. The images were reconstructed using a two-dimensional Filter Back Projection using a Hanning Filter with a Nyquist cutoff at 0.5, and were corrected for attenuation using the Co-57 attenuation scan data. A calibration was conducted to Becquerel per cubic centimeter units using a germanium-68 phantom which was scanned in the Inveon microPET and was reconstructed under the same parameters as the subjects. Analyses of all data were carried out using the Acquisition Sinogram Image Processing IDL's virtual machine (ASIPRO VM) and Pixelwise Modeling software (PMOD 3.0). The test and retest microPET studies on the same animal were carried out within an interval of approximately 2 weeks.

### Metabolite analysis

Blood was collected at four different time points (5, 15, 60, and 90 min) after the injection of ^18^F-nifene. The blood was centrifuged for 5 min at 3,000 g. The plasma was separated and counted. Acetonitrile was added to the blood samples, and the organic layer was spotted on the analytical thin layer chromatography (TLC) plates (silica-coated plates, Baker-Flex, Phillipsburg, NJ, USA) and was developed in 15% methanol in dichloromethane. A sample of the plasma was also collected prior to the injection of ^18^F-nifene and was spiked with the tracer and was used as a standard.

Male Sprague-Dawley rats were injected intravenously (IV) with 0.5 mCi of ^18^F-nifene in a total volume of 0.3 mL and were sacrificed 40 min after injection. The brain was extracted and dissected into two hemispheres. The sagittal sections of 40-μm thickness were obtained from the left hemisphere using the Leica 1850 cryotome and were exposed to phosphor films overnight. The films were read using the Cyclone Phosphor Imaging System and were analyzed using the Optiquant software. The right hemisphere was homogenized with 1.15% KCl (2 mL), and this homogenized mixture was vortexed with 2% acetic acid in methanol (2 mL). This mixture was centrifuged for 10 min at 10,000 g, and the supernatant was removed for analysis. RadioTLC (9:1, dichloromethane and methanol) was obtained for both ^18^F-nifene standard and the brain extract.

### *Ex vivo *microPET

In order to ascertain the brain uptake of ^18^F-nifene, after completion of the *in vivo *microPET scans, the rats were sacrificed and the brain was extracted for *ex vivo *microPET imaging. The whole brain was placed in a hexagonal polystyrene weighing boat (top edge side length, 4.5 cm; bottom edge side length, 3 cm) and was covered with powdered dry ice. This boat was placed securely on the scanner bed, and a transmission scan was acquired. Subsequently, a 60-min emission scan was acquired by the Inveon microPET scanner in full list mode. The list mode was collected in a single frame, and a reconstruction of the images was similar to the procedure described previously in the section "MicroPET ^18^F-nifene studies." The images were analyzed using the ASIPRO VM and PMOD 3.0 software.

### *Ex vivo *autoradiography

The brain after the *ex vivo *microPET acquisition in the section "*Ex vivo *microPET" was removed from the dry ice and was rapidly prepared for sectioning. Horizontal sections (40-μm thick) containing brain regions of the thalamus, subiculum, cortex, striatum, hippocampus, and cerebellum were cut using the Leica CM1850 cryotome. The sections were air-dried and exposed to phosphor films overnight. The films were read using the Cyclone Phosphor Imaging System. The regions of interest of the same size were drawn and analyzed on the brain regions rich in α4β2 nicotinic receptors using the OptiQuant software, and the binding of ^18^F-nifene was measured in digital light units per square millimeter.

### MicroPET studies of nicotine challenge

Nicotine challenge experiments were of two types. In order to demonstrate reversibility of bound ^18^F-nifene and to measure the off-rate, the postinjection nicotine effects were first measured. Sprague-Dawley rats were injected with ^18^F-nifene (0.2 to 0.5 mCi, IV) and at approximately 30 min postinjection of the ^18^F-nifene, 0.3 mg/kg of nicotine free base (administered as a ditartarate salt from Sigma Chemical Company, St. Louis, MO, USA) was administered intravenously. The total time of scan was 90 min and was acquired in full list mode, similar to the protocol for the control scans described in "MicroPET ^18^F-nifene studies." Before and after images were analyzed using the PMOD 3.0 software, and a time-activity curve was generated.

The second set of nicotine challenge experiments were designed to measure α4β2 nAChR receptor occupancy (nAChR_OCC_) by nicotine. Male Sprague-Dawley rats were preinjected intravenously with nicotine using saline for baseline, and four different doses of nicotine (0.02, 0.1, 0.25, and 0.5 mg/kg free base, administered as a ditartarate salt) were diluted in a total volume of 0.3 mL sterile saline. Nicotine was injected 15 min prior to intravenous injection of ^18^F-nifene (0.8-1.0 mCi). Once anesthetized, the rats were scanned for 90 min using the Inveon microPET scanner in full list mode. Dynamic data were reconstructed and analyzed as described in the section "MicroPET ^18^F-nifene studies." Time-activity curves were measured and analyzed using the ASIPRO VM and PMOD 3.0 software. Percent occupancy was calculated from: (Thal_cont _- Thal_nic_/Thal_cont_]) × 100, where Thal_cont _is the percent injected dose of ^18^F-nifene in the brain regions of the control study, and Thal_nic _is the percent injected dose of ^18^F-nifene in the brain regions of the nicotine study at 60 min postinjection of ^18^F-nifene.

## Results

### MicroPET ^18^F-nifene binding studies

**Figure 2:**
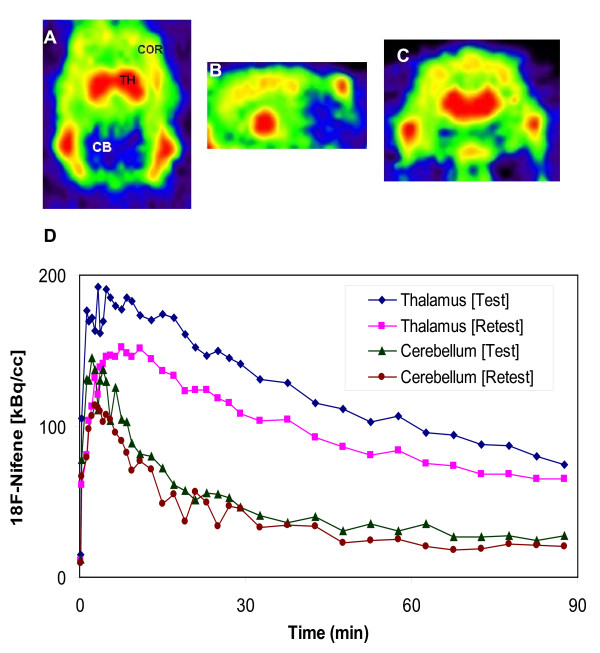
***In vivo *microPET rat brain test-retest study**. **(A) **Horizontal, **(B) **sagittal, **(C) **coronal of ^18^F-nifene. The thalamus (TH) shows the highest binding followed by the cortex (COR) and the cerebellum (CB). Test-retest study showing consistency in binding of ^18^F-nifene to the thalamus with respect to the cerebellum. BP_ND _for the test study was 1.69 while the retest study was 1.64.

A rapid uptake of ^18^F-nifene was observed in the brain with levels of approximately 1% of injected dose per cubic centimeter. Thalamic regions exhibited the highest retention as it has a maximum amount of α4β2 receptors. Significant levels of uptake were observed in the various regions of the cortex while very little binding is present in the cerebellum (Figure 2A,B,C). Time-activity curves of the thalamus, frontal cortex, and cerebellum in Figure 2D show initial rapid uptake in various brain regions followed by greater retention in the thalamus and cortex compared to the cerebellum. A ratio of the uptake for the thalamus and frontal cortex against the reference region cerebellum reached a plateau at approximately 60 min postinjection. The thalamus to cerebellum ratio was approximately 3.5 and the cortex to cerebellum ratio was 2.3.

### Metabolite analysis

**Figure 3:**
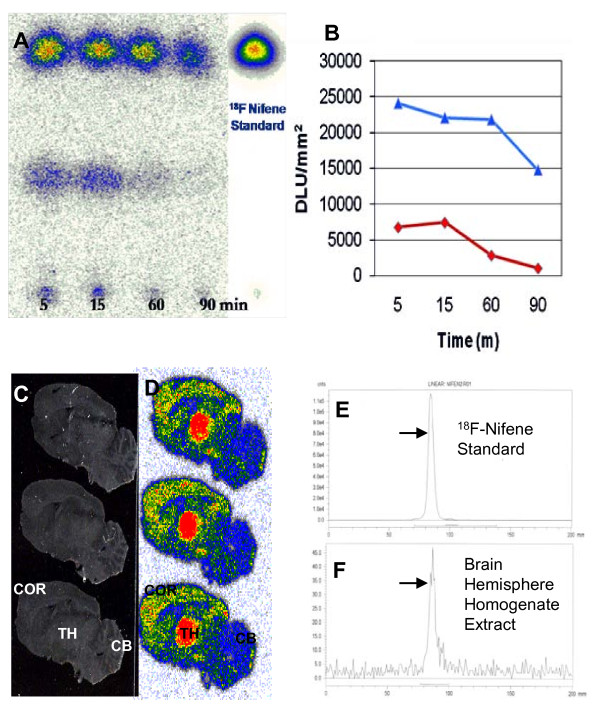
**Blood and brain metabolite analysis in rats postadministration of intravenous ^18^F-nifene**. **(A) **Blood plasma collected at different time points (5, 15, 60, and 90 min) and compared to ^18^F-nifene standard on TLC. A polar metabolite is seen, but the predominant radioactive species is ^18^F-nifene. **(B) **Analysis of TLC in (A) indicates 42% of ^18^F-nifene (blue) remaining at 90 min with little polar metabolites (red) remaining in the plasma. **(C) ***Ex vivo *rat brain was dissected into two hemispheres--the left hemisphere was cut into 40-μm thick sagittal brain sections and were scanned to reveal brain areas. **(D) **Binding of ^18^F-nifene in the thalamus (TH), cortex (COR), and least binding in the cerebellum (CB) was observed. **(E) **RadioTLC of ^18^F-nifene standard with 9:1 CH_2_Cl_2_:CH_3_OH. **(F) **RadioTLC of brain extracts with 9:1 CH_2_Cl_2_:CH_3_OH showing the presence of ^18^F-nifene.

Following the injection of ^18^F-nifene, blood was collected at different time points to measure metabolites in the blood plasma. Figure 3A shows a decrease in the amount of parent as well as metabolites found in the blood plasma during the 90 min. ^18^F-Nifene standard was used to compare the tracer found in the blood plasma. Figure 3B represents about 42% of ^18^F-nifene remaining in the blood plasma at 90 min (compared to that measured at 5 min pi) while the levels of metabolites were significantly reduced in the blood plasma at 90 min.

Radiochromatograms were attained from running brain extracts and were compared to the peak to the parent compound providing evidence that the primary species within the brain of the rat was ^18^F-nifene. After sacrificing the rat, the brain was excised and dissected into the left and right hemispheres. Figure 3C,D shows the sagittal brain slices of the left hemisphere representing the total binding of ^18^F-nifene revealing maximal binding in the thalamus followed by extrathalamic regions such as the cortex and subiculum. The cerebellum had the least amount of activity. A thin layer chromatographic analysis of the extract of the homogenized right hemisphere shown in Figure 3F closely correlates with the retention of ^18^F-nifene standard (Figure 3D). No other significant metabolite peak was observed in the brain extract.

### Test-retest

Test and retest studies were investigated in a group of rats (Figure 2). Binding of ^18^F-nifene in each region of the brain remained consistent among the studies. Figure 2 represents the time-activity curves for a test-retest study in one animal. The curve seen for the retest study follows the same pattern as the test study. By 60 min into the scan, nonspecific binding is seen to be cleared out in both studies and remains at stable levels. The binding potentials for the three rats were calculated and were found to vary between 1.03 and 1.69, but within subject, the test-retest error was approximately 3% (Table 1).

**Table 1:** Test-retest ^18^F-nifene binding potential in thalamus

	Test	Retest	Mean	%Error
Rat 1	1.69	1.64	1.67	3.0%
Rat 2	1.17	1.21	1.19	3.4%
Rat 3	1.06	1.03	1.05	2.9%

Error estimates are given as [(Scan1-Scan2)/(Scan1 + Scan2)/2] × 100

### *Ex vivo *studies

**Figure 4:**
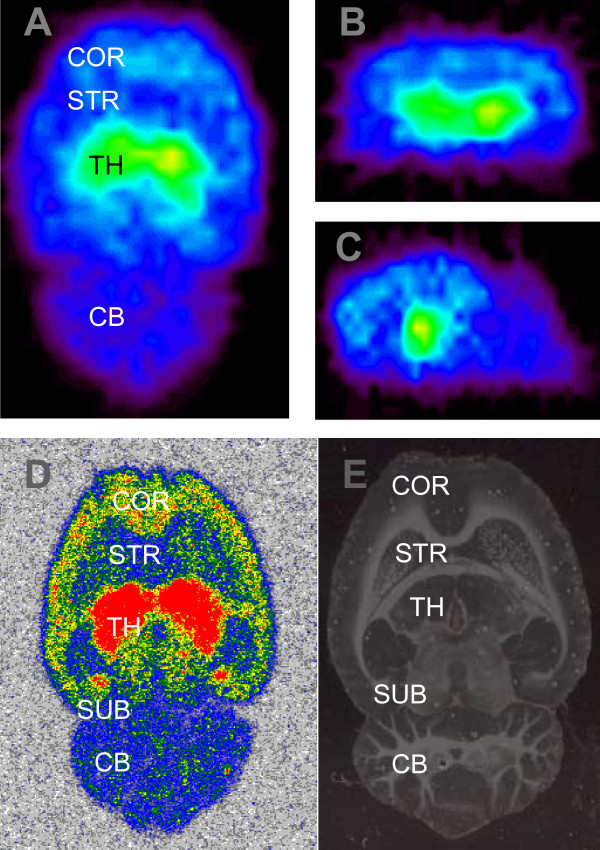
***Ex vivo *microPET and autoradiographic brain images of a rat**. MicroPET images (**(A) **horizontal, **(B) **coronal, and **(C) **sagittal) validate maximum binding in the thalamus (TH) followed by the cortical regions (COR). An autoradiograph of the brain in (A) showing 10-μm horizontal sections **(D) **and an anatomical view **(E) **of the slice in **(D)**. ^18^F-nifene binding followed the order TH > subiculum (SUB) > cortex (COR) > striatum (STR) > cerebellum (CE).

*Ex vivo *microPET imaging of the excised brain after 90 min of *in vivo *scans was carried out for another 60 min. Results clearly show binding of ^18^F-nifene in the thalamus, cortical regions with little binding in the cerebellum (Figure 4A,B,C). This is consistent with the *in vivo *images shown in Figure 2A,B,C.

*Ex vivo *autoradiographs revealed a significant amount of detail that was not readily apparent in the microPET images. The thalamus exhibited the highest amount of ^18^F-nifene binding. The subiculum had a higher amount of binding in the autoradiographs not readily measureable in the microPET data. The cortex had a significant amount of binding consistent to that observed in the microPET imaging data. The cerebellum had the lowest amount of ^18^F-nifene binding in the *ex vivo *autoradiographs. Autoradiographic ratios with respect to the cerebellum in the various brain regions were: thalamus = 4.60, subiculum = 2.39, cortex = 1.83, striatum = 1.46. These ratios are in close agreement to the ratios measures by microPET *ex vivo *(Table 2).

**Table 2:** Measured ^18^F-nifene ratios of rat brain regions with reference to the cerebellum

Brain regions	*In vivo *microPET^a^	*Ex vivo *microPET^b^	*Ex vivo *autoradiographs^c^
Thalamus	3.13 ± 0.29	3.92 ± 0.49	4.60 ± 0.52
Subiculum	-	2.28 ± 0.24	2.39 ± 0.15
Cortex	1.98 ± 0.10	2.05 ± 0.17	1.83 ± 0.19
Striatum	1.52 ± 0.39	1.77 ± 0.28	1.46 ± 0.07

Average of four animals with standard deviations; ^a^Ratio measured at 85-90 min postinjection of ^18^F-nifene; ^b^Ratio measured in the 60-min summed *ex vivo *scan of the same rats; ^c^Ratios measured in sections after the *ex vivo *scans of the same rats.

### MicroPET studies of nicotine challenges

**Figure 5:**
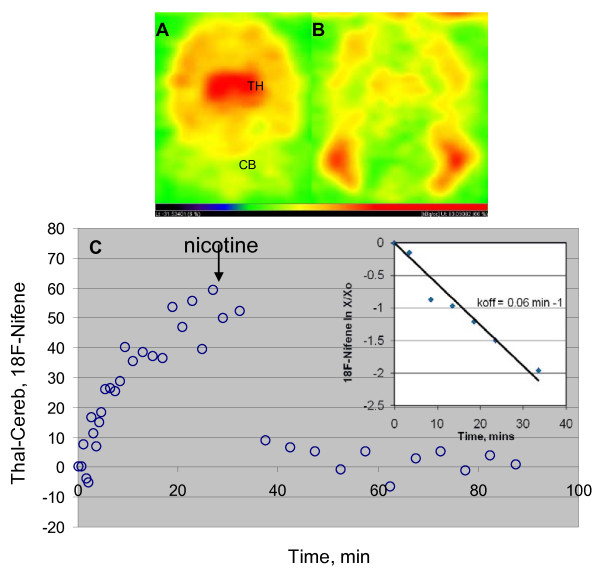
***In vivo *displacement of ^18^F-nifene by nicotine**. *In vivo *rat microPET brain slices of ^18^F-nifene before **(A) **and after **(B) **nicotine challenge. **(C) **Time-activity curve of ^18^F-nifene specific binding (thalamus-cerebellum) with nicotine (0.3 mg/kg) administered at 30 min pi, displacing ^18^F-nifene binding in the thalamus (inset shows dissociation rate, k_off _of ^18^F-nifene was 0.06 min^-1^).

In the first set of experiments with nicotine, ^18^F-nifene bound in the thalamus (Figure 5A) was displaced by IV administration of 0.3 mg/kg of nicotine (Figure 5B). The time-activity curve for this competition of nicotine with ^18^F-nifene in the thalamus is shown in Figure 5C which shows the displacement of most of the ^18^F-nifene from the thalamus. Nicotine had little effect in the cerebellum. The nicotine-induced *in vivo *off-rate measured for ^18^F-nifene was 0.06 min^-1 ^(Figure 5D).

**Figure 6:**
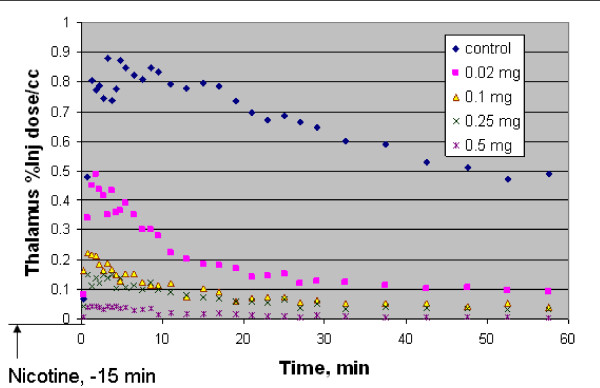
**Dose effects of nicotine on thalamus time-activity curves**. Time-activity curves of ^18^F-nifene uptake in the thalamus of rats injected with different doses of nicotine.

Occupancy of α4β2 nAChR_OCC _by nicotine was measured by dose escalation competition experiments of nicotine with ^18^F-nifene. A change in thalamus binding at baseline was measured at different nicotine doses of injected nicotine. The displacement of ^18^F-nifene was found with the pre-nicotine challenges. With each dose increase of nicotine, a steady increase in binding occupancy was found. The results are summarized in Table 3. Eighty percent binding occupancy was seen with just 0.02 mg/kg of nicotine while 94% binding occupancy was found with 0.5 mg/kg. Figure 6 presents a steady decrease of ^18^F-nifene with the competition of nicotine at different doses.

**Table 3:** Nicotine dose effects on ^18^F-nifene binding

Nicotine, mg/kg	% Injected dose/cc thalamus	Nicotine occupancy
0	0.489	0%
0.02	0.092	81%
0.10	0.037	92%
0.25	0.031	94%
0.50	0.005	99%

Average of two measurements for each dose; receptor occupancy was calculated on the basis of percent injected dose per cubic centimeter of ^18^F-nifene in the thalamus (Thal_cont _- Thal_nic_/Thal_cont _× 100).

## Discussion

Our primary goal was to evaluate ^18^F-nifene binding to the α4β2 receptors in thalamic and extrathalamic brain regions of rodents using microPET imaging. ^18^F-Nifene, an agonist, was developed with fast binding kinetics and a shorter scan time in order to image the α4β2 nicotinic receptors. This is useful in the assessment of nicotinic receptors in neurological diseases. MicroPET studies in rats validated the faster binding profile of ^18^F-nifene thus providing shorter scan times. Maximum binding was found in the thalamus, while moderate binding is seen in the cortex, and minimal binding in the cerebellum. Time-activity curves for the thalamus, cortex, and cerebellum show that ^18^F-nifene peaks early into the scan, and nonspecific binding in the cerebellum cleared rapidly. Thalamus to cerebellum ratios were > 3.0 and cortex to cerebellum were approximately 2. Thus, ^18^F-nifene allows shorter duration PET studies for quantitative measures of α4β2 receptors compared to 2-^18^F-FA-85380 which has been shown to require 5 h to reach steady state in rodents [17].

No lipophilic metabolites of ^18^F-nifene were detected in plasma extracts, and a significant amount of ^18^F-nifene parent remained in the blood after 90 min of the PET study. The absence of lipophilic metabolites was also confirmed using brain extracts of rats injected with ^18^F-nifene. Only ^18^F-nifene was detected in the brain extracts.

The binding of ^18^F-nifene to α4β2 receptors of the rodent brain in microPET studies gave results consistent with the receptor distribution and was comparable with the autoradiographic slices done *in vitro *[3]. Test-retest results of binding potentials, summarized in Table 1, remained consistent between scans thus confirming reproducibility of ^18^F-nifene with <5% standard deviation, suggesting ^18^F-nifene to be suitable for PET studies. *Ex vivo *images, both microPET and autoradiographic, confirmed binding of ^18^F-nifene to thalamic and extrathalamic regions seen in the *in vivo *microPET study.

Nicotine, because of its high affinity to α4β2 receptors, exhibited competition with ^18^F-nifene. Previous *in vitro *studies using 10 nM of nicotine displaced 60-65% in the thalamus region and 300 μM of nicotine, 95% elimination is seen in the thalamus [2]. As expected, displacement of ^18^F-nifene binding was seen in the post-nicotine challenge similar to that reported for 2-[^18^F]F-A-85380 [17]. Figure 6 clearly shows a drop in binding at the time of nicotine injection (30 min into the scan), displacing at least > 80% of ^18^F-nifene binding. The ability for nicotine to compete with ^18^F-nifene can be used to detect changes in receptor occupancy suggesting PET to be a valuable tool in assessing tobacco-related dependence [13]. Pre-nicotine challenges at different dose levels of nicotine, demonstrated a steady decrease in ^18^F-nifene occupancy with respect to nicotine. At low doses of nicotine, 0.02 mg/kg, > 40% of receptors were occupied while at high doses (0.5 mg/kg) > 80% receptors were occupied with nicotine (Table 3). While the cerebellum was used as a reference region, some issues have risen questioning the validity of the cerebellum as a reference region. With the presence of nicotinic receptors in the rat cerebellum [17-19], measurement of binding potential can be complex. Studies using 2-[^18^F]F-A-85380 in rodents have reported nicotine displaceable component in the cerebellum [17], suggesting a need for arterial input function for accurate quantification.

Aside from the cerebellum, efforts have been underway to identify other regions of the brain, such as the corpus callosum and pons as reference regions [20]. Efforts are underway in our rodent ^18^F-nifene studies to identify other reference regions in the brain, other than the cerebellum. Future work in the rodent model will incorporate arterial blood sampling for more accurate quantification.

## Conclusions

^18^F-nifene binds to the α4β2 receptors in thalamic and extrathalamic regions in rat microPET studies. With its faster binding kinetics, short scan time, and reversible binding, ^18^F-nifene is an agonist radiotracer with potential for studying this receptor system in various rodent models.

## Competing interests

The authors declare that they have no competing interests.

## Authors' contributions

MicroPET imaging studies, autoradiographic studies and analysis were carried out by RK and PP, synthesis and metabolite analysis were carried out by SKP and MLP, brain metabolism studies were carried out by BE and JM, microPET data analysis was carried out by CC. The study and all data acquired was coordinated and reviewed by JM. All authors read and approved the final manuscript.
